# Factors influencing vaccination willingness in the context of the COVID-19 pandemic: data from the CoCo-Fakt study

**DOI:** 10.1186/s12889-026-28249-5

**Published:** 2026-06-25

**Authors:** Leon Derakhshani, Sven Feddern, Barbara Grüne, Luis Haberstock, Annelene Kossow, Johannes Niessen, Susanne Rost, Gerhard A. Wiesmüller, Nikola Schmidt, Christine Joisten

**Affiliations:** 1 Department of Infection Control and Environmental Hygiene, Cologne Health Authority, Cologne, Germany; 2 Formerly Department of Infection Control and Environmental Hygiene, Cologne Health Authority, Cologne, Germany; 3 Institute of Hygiene, University Hospital Muenster, Münster, Germany; 4 Institute for Occupational Medicine and Social Medicine, University Hospital, Medical Faculty, RWTH Aachen University, Aachen, Germany; 5 Department for Physical Activity in Public Health, German Sport University Cologne, Institute of Movement and Neurosciences, Am Sportpark Müngersdorf 6, Cologne, 50933 Germany; 6 Augsburg County Health Authority, Augsburg, Germany

**Keywords:** Public health, Vaccine acceptance, Vaccine hesitancy, Covid, Health literacy

## Abstract

**Background:**

During the COVID-19 pandemic, the population’s willingness to be vaccinated was a decisive factor in containing infections. International studies have identified that health literacy and sociodemographic characteristics play a significant role in vaccination decisions. This study investigated possible factors influencing vaccination willingness in Germany.

**Methods:**

Data were collected from the CoCo-Fakt study, which used an online questionnaire to survey infected cases and contact persons from the health authorities in Cologne and Augsburg county. Sociodemographic data and information on chronic diseases, vaccination status, willingness to be vaccinated, and subjectively perceived health literacy via a modified HLS19-Q47 questionnaire were collected. A total of 9,705 people was included in the analysis. Factors associated with vaccination willingness were assessed using chi-squared-tests and t-tests, followed by binary logistic regression with backward elimination to identify independent associations.

**Results:**

Of those surveyed, 91.6% had already been vaccinated against COVID-19 or were willing to be vaccinated, while 8.4% had refused. A higher willingness to be vaccinated was found among older people (OR = 1.02), infected individuals (OR = 1.98), individuals with chronic diseases (OR = 1.32), individuals with higher socioeconomic status (OR = 1.26), and those with high health literacy (OR = 1.28). By contrast, individuals with a migration background (OR = 0.39) and those with moderate health literacy (OR = 0.76) showed greater reluctance to be vaccinated.

**Conclusion:**

The results underscore the importance of individual and social factors for vaccination acceptance. Particularly vulnerable groups included younger adults, individuals with a migration background, and those with moderate health literacy. This highlights the need to tailor future vaccination campaigns to target specific groups, possibly through low-threshold, multilingual information services and targeted health literacy promotion.

## Background

In December 2019, the first cases of a novel coronavirus disease, coronavirus disease 2019 (COVID-19), appeared in Wuhan, China, caused by SARS-CoV-2 [1]. Its rapid global spread led the World Health Organization (WHO) to declare a pandemic on 11 March 2020 [2]. Because no specific therapeutic options or vaccines were available in the early stages, measures such as contact restrictions and curfews were relied upon initially [3]. However, the approval of the first COVID-19 vaccine (Comirnaty from BioNTech/Pfizer) in December 2020 provided a crucial tool to effectively reduce infection rates and severe disease progression [4, 5].

One year after the start of the programme, 62.8 million people in Germany had been vaccinated at least once, but a significant group remained unwilling [6]. Previous research has identified several determinants of vaccination willingness, including sociodemographic, health-related, socioeconomic, and attitudinal factors. Younger age, female sex, lower educational attainment, migration background, and lower socioeconomic status have been associated with reduced vaccine uptake or willingness in several studies [7–10]. In contrast, chronic disease has been linked to higher vaccination rates, possibly reflecting greater perceived vulnerability to severe COVID-19 [11, 12].

In addition, health literacy has also been discussed as an influencing factor. Health literacy is defined as the ability to find, understand, and critically evaluate health-related information and apply it to decision-making [13]. Some studies have suggested that individuals with limited health literacy may face greater difficulty in assessing the benefits and risks of vaccination, navigating complex health information, and distinguishing reliable from unreliable sources, which may in turn affect their vaccination decisions. However, the evidence to date is predominantly based on online survey data from specific populations, such as students or volunteers [14–16]. In addition, these studies have often addressed either vaccination status or willingness to be vaccinated, but rarely both. To date, few studies have been conducted in Germany, with existing studies mainly originating from Africa or Asia.

Therefore, this study examined possible factors influencing vaccination willingness in the context of the COVID-19 pandemic. By analysing a large regional sample of directly affected individuals, the results contribute to the development of potential practice-relevant starting points for vaccination strategies.

## Methods

From the end of February 2020, individuals residing in Cologne and Augsburg with SARS-CoV-2 infection (infected persons, IPs), as detected by quantitative real-time polymerase chain reaction (PCR) or rapid testing, were reported to the respective health authorities, along with their contact persons (CPs). In Cologne, a database of these details was developed by the City of Cologne’s Office for Information Processing and the Cologne Health Authority, called DiKoMa (Digital Contact Management) [17]. In accordance with the Infection Protection Act (IfSG), those affected were placed in isolation or quarantine for the applicable period. Similarly, in the district of Augsburg, IPs and CPs were entered into a database and placed in isolation or quarantine.

Participants were required to be at least 16 years old and to provide a declaration of consent and an email address. Exclusion criteria were lack of compliance, deceased persons, and persons in medical or nursing facilities, and false positives (i.e. reported SARS-CoV-2 infections later found not to be confirmed). At the beginning of the questionnaire, information about the background of the study and data protection was provided.

Reported IPs and CPs received an email invitation from their respective authority asking them to participate in the CoCo-Fakt monitoring study, a mixed-methods online survey conducted in three waves in Cologne (Waves 1–3) and district of Augsburg (Wave 3). In each round of surveys, email addresses were removed from the data to ensure anonymity, so that responses could not be traced to individual respondents. Reminders were sent two and four weeks after the invitation.

In Wave 1 (survey from 12 December 2020 to 6 January 2021), 36,498 individuals were contacted, and 11,346 responded to the questionnaire (data not presented here). In Wave 2 (survey from 26 July 2021 to 8 September 2021), 37,532 people were contacted, and 5,970 responded. In Wave 3 (survey from 30 May 2022 to 16 October 2022), 105,376 people were contacted, and 5,830 responded.

This study includes data from the second and third waves as vaccinations were not available during the first wave. The questionnaire required approximately 30–45 min to complete and was conducted using Unipark software [18].

### Questionnaire

The questionnaire was based on the COVID-19 Snapshot Monitoring Study conducted by the University of Erfurt and the WHO and modified to align with the target questions [19, 20]. The questions used for this analysis were as follows:

#### Sociodemographic data

Participants were asked about their age (in years), sex (male/female), language predominantly spoken at home, and highest education level and professional qualifications. An additional question addressed employment status (full-time, part-time, or occasional/irregular/unemployed). Language was used to classify migration background, with German indicating no migration background and any other language indicating a migration background. The SES was calculated based on school education and occupation, using a modified version of the method of the German Health Interview and Examination Survey for Adults (DEGS1) [21]. We did not collect data on income; instead, we scaled the metrics for the remaining two dimensions accordingly and classified them into ‘high’, ‘medium’, and ‘low’ using the DEGS1 methodology. However, due to the very small proportion of participants classified as having low SES (1.4%), the low and medium categories were combined into a single group (“low/medium”) to ensure sufficient cell sizes for stable statistical analyses.

#### Health status

Participants were asked about the presence of chronic diseases, weight, and height. BMI and related classifications were determined according to WHO guidelines [22].

#### Vaccination status

Participants were asked about their COVID-19 vaccination and infection history, as well as their vaccination willingness. The answers were summarised using the following variables:

- *Willingness to be vaccinated/Vaccine acceptance: I have been vaccinated or would like to be vaccinated.*
- *Non-willingness to be vaccinated/Vaccine hesitancy: No*,* I do not want to be vaccinated.*

#### Reason for quarantine

Each respondent’s reason for quarantine or isolation was recorded as IP or CP.

#### Health literacy

Health literacy was assessed using a modified version of the HLS19-Q47, as used in the HLS-GER2 study [23], with the following question rated on a six-point Likert scale (‘very easy’ to ‘very difficult’):

*How easy/difficult is it for you to…*.

- ...understand the quarantine measures communicated to you by the health authority?
- ...understand why it is important to remain in quarantine as a contact person?
- ...understand what the health authority or doctor tells you?
- ...follow the instructions of your health authority on how to behave during quarantine?
- ...understand health advice for family members or friends during the quarantine period?
- ...find information about support options for mental health issues during quarantine?
- ...find information about measures you should take during quarantine?
- ...assess which activities would be good for you during quarantine (or, if applicable, coronavirus infection)?
- ...find information about activities that could be good for your mental well-being during quarantine? (e.g., activities such as meditation, physical exercise, Pilates, etc.)
- ...apply the information or activities that you found useful to yourself?
- ...understand information in the media about how you can improve your health during quarantine (or, if applicable, coronavirus infection)? (e.g., from the internet, newspapers, magazines)
- ...assess whether the information about coronavirus (infections) in the media is trustworthy? (e.g., from television, the internet or other media)
- ...understand health warnings about certain behaviours during quarantine, such as smoking and unhealthy eating?
- ...assess which everyday habits are related to your health and recovery? (Drinking and eating habits, exercise, etc.)
- ...find information about (coronavirus) disease symptoms that may affect you?
- ...find information about measures that could be helpful in the context of a coronavirus infection?
- ...find out where you can get professional help for a coronavirus infection? (Doctor, pharmacist, psychologist)
- ...assess when you should contact a doctor?
- ...find information about what to do in the event of a medical emergency?

The items were then recoded (1 = ‘very difficult’, 6 = ‘very easy’), and the values were summed and divided by the number of items completed. Tertiles were formed from the total values of the sample, and health literacy was classified as low (1 to < 3.18), medium (3.18 to < 4.89), or high (4.89 to 6).

### Data analysis

Data analysis was performed using SPSS 29.0. Univariate differences were examined using chi-squared tests and t-tests. Effect sizes were calculated using Cohen’s d (independent t-test; trivial < 0.2; small 0.2–0.5; medium 0.5–0.8; large ≥ 0.8), Cramer’s V (chi-squared test; small 0.06–0.15; medium: 0.16–0.26; large > 0.26), and Eta-squared (ANOVA; small: 0.01–0.05; medium: 0.06–0.13; large: ≥ 0.14) [24].

Binary logistic regression was used to examine possible factors influencing willingness to be vaccinated (no = 0, yes = 1). The following variables were included in the model: reason for quarantine (IP = 0, CP = 1); sex (female = 1, male = 2); age (in years), migration background (no = 0, yes = 1); SES (low/medium = 0, high = 1); chronic diseases (no = 0, yes = 1); and health literacy (low = 1, medium = 2, high = 3). Variables that did not contribute to the regression equation were removed by backward elimination. The significance level was set at α = 0.05.

## Results

### Study population

**Fig. 1:**
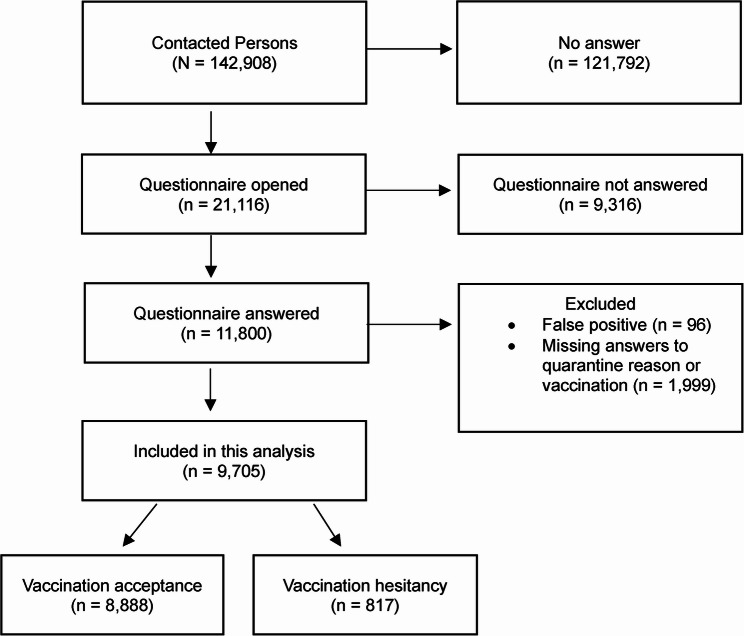
Flowchart of the selection of the study population from the CoCo-Fakt survey. The data were collected in the second (July-September 2021) and third (May-October 2022) waves of the CoCo-Fakt study

A total of 9,705 individuals were included in the analysis (Fig. 1); 60.3% were women (*n* = 5,854) and 39.7% were men (*n* = 3,851; Table 1). The respondents were between 16 and 99 years old, with an average age of 42.3 ± 14.1 years. The average self-reported weight was 77.9 ± 18.2 kg, the average height was 172.5 ± 9.3 cm, and the average BMI was 26.1 ± 5.3 kg/m²; 1.9% of participants (*n* = 180) were underweight, 47.3% (*n* = 4,483) were of normal weight, 32.6% (*n* = 3,088) were overweight, and 18.2% (*n* = 1,729) were obese. Chronic diseases were reported by 25.9% of respondents (*n* = 2,504). The proportion of individuals with a migration background was 8.2% (*n* = 787). The majority (63.9%; *n* = 5,592) were classified as having high SES, with 36.1% (*n* = 3,166) classified as having low or medium SES. Most respondents were employed, with 57.3% (*n* = 5,536) in full-time and 25.6% (*n* = 2,471) in part-time positions; 17.1% (*n* = 1,647) were employed in occasional or irregular employment or unemployed.

**Table 1:** Sociodemographic and health characteristics of the study population (*n* = 9,705), stratified by sex

		Total *n* = 9,705	Female *n* = 5,854	Male *n* = 3,851	*p*-Value	Effect size
Age (years)		42.3 ± 14.1	40.9 ± 13.6	44.5 ± 14.6	<.001ᵇ	0.256ᵃ
Height (cm)		172.5 ± 9.3	167.3 ± 6.5	180.4 ± 7.0	<.001ᵇ	1.961ᵃ
Weight (kg)		77.9 ± 18.2	71.2 ± 16.2	87.9 ± 16.2	<.001ᵇ	1.029ᵃ
BMI (kg/m²)		26.1 ± 5.3	25.4 ± 5.6	27.0 ± 4.6	<.001ᵇ	0.296ᵃ
BMI classification, n (row %)					<.001ᵈ	0.213ᶜ
	Underweight	180	160 (88.9)	20 (11.1)		
	Normal weight	4,483	3,093 (69.0)	1,390 (31.0)		
	Overweight	3,088	1,469 (47.6)	1,619 (52.4)		
	Obese	1,729	954 (55.2)	775 (44.8)		
Reason for quarantine, n (row %)					.502ᵈ	
	IP	7,154	4,301 (60.1)	2,853 (39.9)		
	CP	2,551	1,553 (60.9)	998 (39.1)		
Migration background, n (row %)					.049ᵈ	0.020ᶜ
	No	8,760	5,301 (60.5)	3,459 (39.5)		
	Yes	787	448 (56.9)	339 (43.1)		
SES, n (row %)					.009ᵈ	0.028ᶜ
	Low/medium	3,166	1,963 (62.0)	1,203 (38.0)		
	High	5,592	3,309 (59.2)	2,283 (40.8)		
Employment, n (row %)					<.001ᵈ	0.352ᶜ
	Full-time	5,536	2,617 (47.3)	2,919 (52.7)		
	Part-time	2,471	2,177 (88.1)	294 (11.9)		
	Occasional/irregular employment or unemployed	1,647	1,032 (62.7)	615 (37.3)		
Chronic diseases, n (row %)					.001ᵈ	0.034ᶜ
	No	7,175	4,256 (59.3)	2,919 (40.7)		
	Yes	2,504	1,580 (63.1)	924 (36.9)		

Values are presented as mean ± standard deviation. For categorical variables, the total column shows absolute numbers; percentages in the sex-stratified columns are row percentages. Effect sizes are reported as Cohen’s dᵃ (t-testsᵇ) or Cramer’s Vᶜ (chi-squared testsᵈ)

*BMI* Body mass index, *CP* Contact person, *IP* Infected person, *SES* Socioeconomic status, *cm* Centimetres, *kg* Kilograms, *m* Metres

Men were older on average than women, at 44.5 ± 14.6 years (*p* <.001, Cohen’s d = 0.256) compared to 40.9 ± 13.6 years. They were also taller and heavier, at 180.4 ± 7.0 cm (*p* <.001, Cohen’s d = 1.961) and 87.9 ± 16.2 kg (*p* <.001, Cohen’s d = 1.029), with a higher BMI (27.0 ± 4.6 kg/m² vs. 25.4 ± 5.6 kg/m²; *p* <.001, Cohen’s = 0.296). Accordingly, they were significantly more likely to be overweight or obese (63.0%, *n* = 2,394 vs. 42.7%, *n* = 2,423; *p* <.001, Cramer’s V = 0.213). Men were more likely to have a high SES (65.5%; *n* = 2,283) than women (62.8%, *n* = 3,309; *p* =.009, Cramer’s V = 0.028; see also Table 1). While women and men were similarly likely to be unemployed, irregularly employed, or occasionally employed, 76.3% of men (*n* = 2,919) and only 44.9% of women (*n* = 2,617) reported working full-time. Conversely, 37.4% of women (*n* = 2,177) and only 7.7% of men (*n* = 294) reported working part-time (*p* <.001, Cramer’s V = 0.352).

### Vaccination status and willingness

Most respondents had already been vaccinated against COVID-19 at the time of the survey (*n* = 7,417, 76.4%). Among them, 24.5% (*n* = 2,374) had received a booster shot, 29.8% (*n* = 2,894) had received two doses, and 7.2% (*n* = 701) had received one dose. A further 14.9% (*n* = 1,448) had been both vaccinated and infected. Meanwhile, 15.2% (*n* = 1,471) of respondents were unvaccinated but expressed a willingness to be, and 8.4% (*n* = 817) stated that they were not vaccinated and did not want to be. In summary, 91.6% (*n* = 8,888; Table 2) of respondents were either vaccinated or willing to be vaccinated, while 8.4% (*n* = 817) refused vaccination.

**Table 2:** Sociodemographic and health characteristics of participants by vaccination willingness

		Vaccination acceptance *n* = 8,888	Vaccination hesitancy *n* = 817	*p*-Value	Effect size
Sex, *n* (row %)				.131ᵈ	
	Female	5,341 (91.2)	513 (8.8)		
	Male	3,547 (92.1)	304 (7.9)		
Age (years)		42.6 ± 14.2	38.7 ± 12.6	<.001ᵇ	0.280ᵃ
Height (cm)		172.6 ± 9.3	171.7 ± 9.3	.007ᵇ	0.100ᵃ
Weight (kg)		78.1 ± 18.2	75.9 ± 17.9	.001ᵇ	0.119ᵃ
BMI (kg/m²)		26.1 ± 5.3	25.7 ± 5.2	.030ᵇ	0.081ᵃ
BMI classification, n (row %)				.044ᵈ	0.029ᶜ
	Underweight	161 (89.4)	19 (10.6)		
	Normal weight	4,078 (91.0)	405 (9.0)		
	Overweight	2,856 (92.5)	232(7.5)		
	Obese	1,598 (92.4)	131 (7.6)		
Reason for quarantine, n (row %)				<.001ᵈ	0.066ᶜ
	IP	6,474 (90.5)	680 (9.5)		
	CP	2,414 (94.6)	137 (5.4)		
Migration background, n (row %)				<.001ᵈ	0.102ᶜ
	No	8,101 (92.5)	659 (7.5)		
	Yes	647 (82.2)	140 (17.8)		
SES, n (row %)				.006ᵈ	0.030ᶜ
	Low/medium	2,873 (90.7)	293 (9.3)		
	High	5,169 (92.4)	423 (7.6)		
Employment, n (row %)				.291ᵈ	
	Full-time	5,089 (91.9)	447 (8.1)		
	Part-time	2,262 (91.5)	209 (8.5)		
	Occasional, irregular, or unemployed	1,494 (90.7)	153 (9.3)		
Chronic diseases, n (row %)				<.001ᵈ	0.039ᶜ
	No	6,526 (90.9)	649 (9.1)		
	Yes	2,340 (93.5)	164 (6.5)		

Values are presented as mean ± standard deviation for continuous variables and n (row %) for categorical variables. Row percentages are calculated across comparison groups within each category. Effect sizes are reported as Cohen’s dᵃ (t-testsᵇ) or Cramer’s Vᶜ (chi-squared testsᵈ)

*BMI* Body mass index, *CP* Contact person, *IP* Infected person, *SES* Socioeconomic status, *cm* Centimetres, *kg* Kilograms, *m* Metres

Respondents who did not want to be vaccinated were younger on average (38.7 ± 12.6 years; *p* <.001, Cohen’s d = − 0.280; Table 2) than those who were willing to be vaccinated (42.6 ± 14.2 years). Participants willing to be vaccinated had a higher average BMI (26.1 ± 5.3 kg/m²; *p* =.030, Cohen’s d = − 0.081) than those who were unwilling to be vaccinated (25.7 ± 5.2 kg/m²). Regarding BMI, respondents who were categorised as pre-obese (*n* = 2,856, 92.5%) or obese (*n* = 1,598, 92.4%) were more likely to be willing to be vaccinated than those with normal weight (*n* = 4,078, 91.0%) or underweight (*n* = 161, 89.4%) (*p* =.044, Cramer’s V = 0.029). Participants with a migration background (*n* = 140, 17.8%; *p* <.001, Cramer’s V = 0.102) were less likely to be willing to be vaccinated than participants without a migrant background (*n* = 659, 7.5%). Respondents with high SES were more willing to be vaccinated (*n* = 5,169, 92.4%; *p* =.006, Cramer’s V = 0.030) than respondents with low or medium SES (*n* = 2,873, 90.7%). Finally, participants with chronic diseases (*n* = 2,340, 93.5%; *p* =.002, Cramer’s V = 0.037) were more willing to be vaccinated than those without (*n* = 6,526, 91.0%). No differences were observed between sexes (*p* =.131) or employment statuses (*p* =.291).

### Health literacy

Health literacy was low in 48.2% (*n* = 4,347) of respondents, medium in 28.0% (*n* = 2,543), and high in 23.9% (*n* = 2,167; Table 3). Women were more likely to have high health literacy than men (25.1%, *n* = 1,378 vs. 22.0%, *n* = 789; *p* =.001, Cramer’s V = 0.039), and men were more likely to have low health literacy than women (50.2%, *n* = 1,802 vs. 46.8%, *n* = 2,572). Medium health literacy was almost equally common in both sexes (women 28.1%, *n* = 1,545; men 27.8%, *n* = 998).

**Table 3:** Sociodemographic and health characteristics of participants by health literacy level (low, medium, high)

		Low HL *n* = 4,374	Medium HL *n* = 2,543	High HL *n* = 2,167	*p*-Value	Effect size
Sex, *n* (row %)					.001ᵈ	0.039ᶜ
	Female	2,572 (46.8)	1,545 (28.1)	1,378 (25.1)		
	Male	1,802 (50.2)	998 (27.8)	789 (22.0)		
Age (years)		42.6 ± 14.5	40.8 ± 13.4	44.9 ± 13.8	<.001ᵇ	0.011ᵃ
Height (cm)		173.0 ± 9.3	172.5 ± 9.3	171.8 ± 8.9	<.001ᵇ	0.003ᵃ
Weight (kg)		78.1 ± 18.3	78.3 ± 18.2	77.3 ± 18.1	.142ᵇ	
BMI (kg/m²)		26.0 ± 5.3	26.2 ± 5.2	26.1 ± 5.4	.255ᵇ	
BMI classification, n (row %)					.383ᵇ	
	Underweight	76 (46.1)	39 (23.6)	50 (30.3)		
	Normal weight	2,038 (48.5)	1,167 (27.8)	998 (23.7)		
	Overweight	1,363 (47.3)	808 (28.0)	713 (24.7)		
	Obese	780 (47.5)	478 (29.1)	383 (23.3)		
Reason for quarantine, n (row %)					<.001ᵈ	0.320ᶜ
	Infected person	2,633 (39.0)	2,134 (31.6)	1,989 (29.4)		
	Contact person	1,741 (74.8)	409 (17.6)	178 (7.6)		
Migration background, n (row %)					<.001ᵈ	0.076ᶜ
	No	3,932 (47.6)	2,333 (28.2)	1,996 (24.2)		
	Yes	411 (61.4)	159 (23.8)	99 (14.8)		
SES, n (row %)					<.001ᵈ	0.153ᶜ
	Low/medium	1,124 (37.5)	971 (32.4)	906 (30.2)		
	High	2,787 (53.1)	1,355 (25.8)	1,108 (21.1)		
Employment, n (row %)					<.001ᵈ	0.064ᶜ
	Full-time	2,557 (49.5)	1,468 (28.4)	1,146 (22.2)		
	Part-time	974 (41.6)	702 (30.0)	667 (28.5)		
	Occasional, irregular, or unemployed	821 (53.6)	364 (23.8)	347 (22.6)		
Chronic diseases, n (row %)					.635ᵈ	
	No	3,228 (48.3)	1,851 (27.7)	1,599 (23.9)		
	Yes	1,133 (47.5)	685 (28.7)	567 (23.8)		
Vaccination status, n (row %)					<.001ᵈ	0.058ᶜ
	Vaccination acceptance	4,045 (48.5)	2,272 (27.2)	2,025 (24.3)		
	Vaccination hesitancy	329 (44.3)	271 (36.5)	142 (19.1)		

Values are presented as mean ± standard deviation for continuous variables and n (row %) for categorical variables. Row percentages are calculated across comparison groups within each category. Effect sizes are reported as Eta-squaredᵃ (ANOVAᵇ) or Cramer’s Vᶜ (chi-squared testsᵈ). Totals differ from overall sample size (*n* = 9,705) because HL data were available for *n* = 9,084 participants

*HL* Health literacy, *BMI* Body mass index, *CP* Contact person, *IP* Infected person, *SES* Socioeconomic status, *cm* Centimetres, *kg* Kilograms, *m* Metres

IPs had significantly higher health literacy than CPs (29.4%, *n* = 1,989 vs. 7.6%, *n* = 178; *p* <.001, Cramer’s V = 0.320).

Participants without a migration background were more likely to have high health literacy than those with a migration background (24.2%, *n* = 1,996 vs. 14.8%, *n* = 99; *p* <.001, Cramer’s V = 0.076).

Respondents with low or medium SES were more likely to have high health literacy than those with high SES (30.2%, *n* = 906 vs. 21.1%, *n* = 1,108; *p* <.001, Cramer’s V = 0.153).

On average, participants with medium health literacy were younger than those with high health literacy (40.8 ± 13.4 years vs. 44.9 ± 13.8 years; *p* <.001, Eta-squared = 0.011).

### Regression analyses

**Fig. 2:**
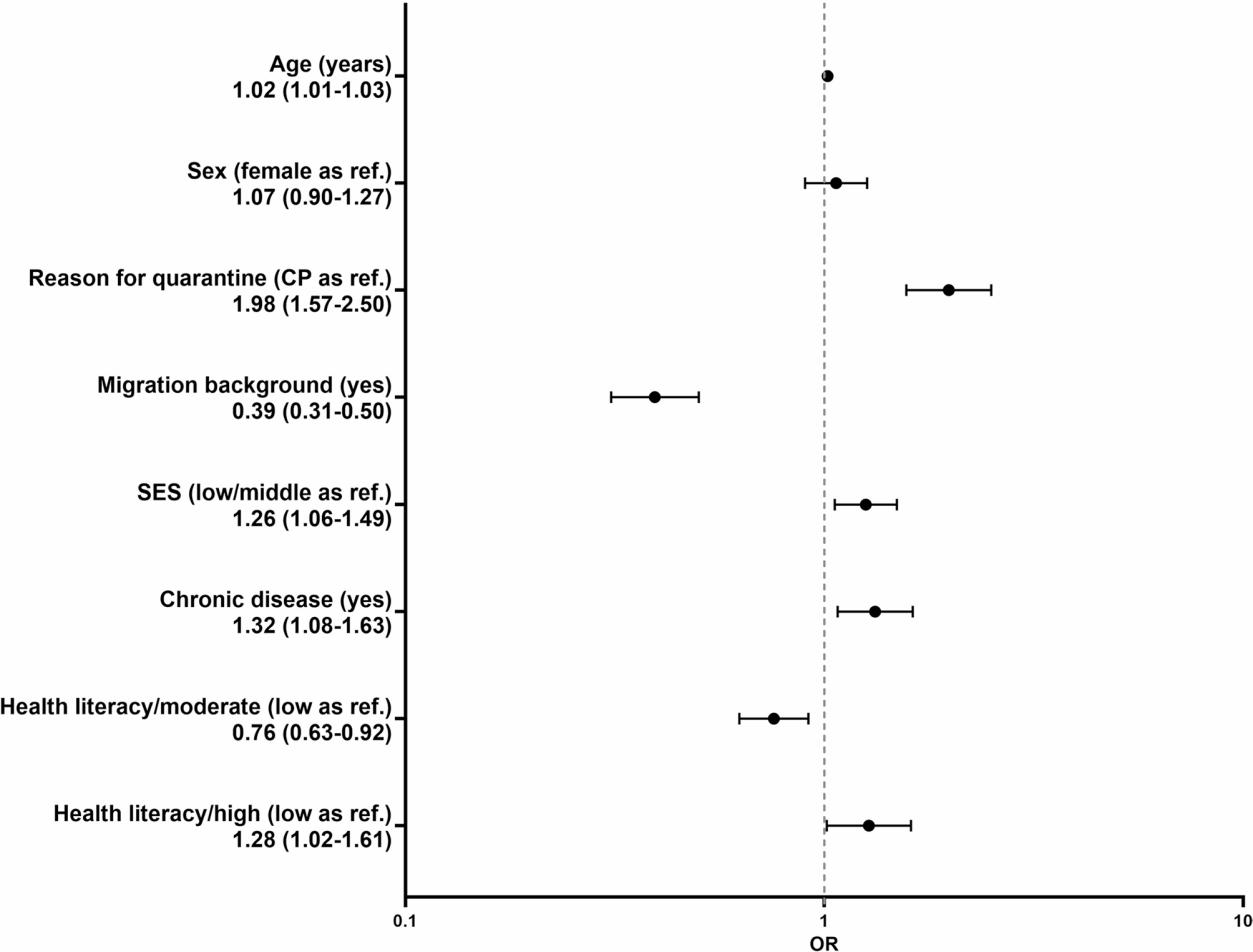
Forest plot of factors influencing the willingness to be vaccinated. Abbreviations: ref = reference; CP = contact person; SES = socioeconomic status

The binary logistic regression analysis showed that older age (*p* <.001, OR = 1.02, 95% CI: 1.01–1.03), higher SES (*p* =.008, OR = 1.26 95% CI: 1.06–1.49), the presence of chronic diseases (*p* =.008, OR = 1.32, 95% CI: 1.08–1.63), being infected (*p* <.001, OR = 1.98, 95% CI: 1.57–2.50) and high health literacy (*p* =.037, OR = 1.28, 95% CI: 1.02–1.61) were each significantly associated with increased willingness to be vaccinated (Table 4). Migration background (*p* <.001, OR = 0.39, 95% CI: 0.31–0.50) and medium health literacy (*p* =.004, OR = 0.76, 95% CI: 0.63–0.92) were associated with a lower willingness to be vaccinated. No significant association was found for sex. The model explained 5.4% of the variance in willingness to be vaccinated (Fig. 2).

**Table 4:** Binary logistic regression of factors associated with willingness to be vaccinated. Odds ratios (ORs) with 95% confidence intervals (CIs) are shown

	B	Standard error	*p*-Value	OR	95% CI	
					Lower	Upper
Age (years)	0.018	0.003	< 0.001	1.02	1.01	1.03
Sex (ref. female)	0.066	0.087	0.452	1.07	0.90	1.27
IP vs. CP (ref. CP)	0.685	0.119	< 0.001	1.98	1.57	2.50
Migration background (ref. no)	−0.930	0.123	< 0.001	0.39	0.31	0.50
SES (ref. low/medium)	0.229	0.087	0.008	1.26	1.06	1.49
Chronic diseases (ref. no)	0.280	0.105	0.008	1.32	1.08	1.63
Medium HL (ref. low)	−0.277	0.097	0.004	0.76	0.63	0.92
High HL (ref. low)	0.246	0.118	0.037	1.28	1.02	1.61

*OR* Odds ratio, *CP* Contact person, *IP* Infected person, *SES* Socioeconomic status, *HL* Health literacy

## Discussion

To our knowledge, this is one of the first studies to examine factors influencing the willingness to be vaccinated during the COVID-19 pandemic in Germany. At the time of the survey, 76% of respondents had already been vaccinated against COVID-19, 15% intended to be vaccinated, and 8% of respondents refused vaccination. This proportion is slightly higher overall than the nationwide vaccination rates of 67% on 8 September 2021 and 77% on 16 October 2022 [6]. While this may indicate a positively selected sample, the data still allow a nuanced view of influencing factors. Older people, those with chronic illnesses, respondents with higher socioeconomic status, individuals who had been quarantined as an infected person rather than as a contact person and those with high health literacy were significantly more likely to be willing to be vaccinated. In contrast, younger participants, individuals with a migration background, and those with medium health literacy showed an increased reluctance to be vaccinated.

Notably, the reason for quarantine was the strongest predictor in the regression model, with infected persons showing nearly twice the odds of vaccination willingness compared to contact persons. This finding may be interpreted in light of protection motivation theory, which posits that direct experience with a health threat can heighten perceived severity and vulnerability, thereby increasing motivation for protective behaviors such as vaccination [25]. However, the relationship between prior infection and vaccination willingness is not unequivocal. In a large US survey of 63,266 adults, Nguyen et al. (2022) found that vaccination receipt was significantly lower among those with a previous COVID-19 diagnosis (aPR = 0.88) [26]. Similarly, Do and Frank (2022) reported that prior infection halved the odds of vaccine receipt in the US, potentially driven by a perceived sufficiency of natural immunity [27]. The divergent direction of this association across studies may depend on factors such as symptom severity, timing relative to the infection, and the prevailing public discourse on natural versus vaccine-induced immunity. In the present study, the higher vaccination willingness among IPs may reflect the specific context of health authority-supervised quarantine, in which infected individuals received direct medical guidance and may have been more receptive to vaccination recommendations.

The observation that older age and chronic illnesses are positively associated with willingness to be vaccinated is consistent with international studies [7, 28]. A cross-sectional study by the European Commission with 23,606 participants found that vaccination scepticism was also 4 times higher among 21- to 39-year-olds and 2 times higher among 40- to 60-year-olds compared to those over 65 [7]. Similarly, a US survey conducted in April 2021 with 2,021 participants showed the lowest vaccine hesitancy among people over 70 (OR = 0.10) and 60- to 69-year-olds (OR = 0.38) compared to 18- to 29-year-olds [28].

People with chronic diseases also showed a greater willingness to be vaccinated. In a systematic review with 12,508 participants across five studies, Nindrea et al. (2021) reported an almost 1.5-fold increase in willingness to be vaccinated compared to healthy individuals [29]. The subjective perception of disease risk is thought to significantly influence motivation to be vaccinated [30]. However, this association may not be uniform across disease types. In a US cross-sectional survey of 2,535 participants, participants with chronic lung disease were more likely to show vaccination acceptance than people without chronic diseases, while people with autoimmune diseases were more ambivalent [11]. The main reasons given by those with autoimmune diseases for not being vaccinated against COVID-19 were concerns about vaccine safety; the primary reason given for being vaccinated was an increased need to protect themselves. By contrast, in a European study of 49,253 participants using cross-sectional telephone data from June to August 2021 (the ninth wave of the Survey of Health, Ageing and Retirement in Europe), those with chronic lung disease and high blood pressure showed reduced willingness to be vaccinated [12], while those with hypercholesterolaemia showed increased willingness. The authors did not speculate on an explanation for these differences but called for further research.

Our data also indicate that sociodemographic characteristics, including migration background, influence willingness to be vaccinated. Limited language skills often hinder access to valid information, can lead to misinterpretations, and are frequently associated with lower health literacy [31]. Low health literacy, mistrust of medical personnel, and lack of access to health information are significantly associated with vaccine hesitancy. Frustration and low self-confidence in searching for health information are likewise linked to greater vaccine scepticism [32].

However, medium levels of health literacy, rather than low levels, were associated with the greatest reluctance to vaccinate. These individuals may be uncertain about how to deal with information and more susceptible to misinformation, whereas people with lower health literacy are more likely to trust and accept health information from television or social media [33], although this remains speculative. In general, people with high health literacy have the skills to systematically search for health information, adequately evaluate it, and apply it to their situation [13]. In our analysis, participants with high health literacy showed the highest willingness to be vaccinated, consistent with previous literature. In a prospective cohort study of 1,647 participants in France, Montagni et al. (2021) reported that people with low health literacy were more likely to show reluctance to be vaccinated than those with high health literacy (OR = 1.44) [15]. In a cross-sectional study of 3,360 participants in Slovenia, Lubej et al. (2025) also found that high health literacy has a positive influence on attitudes towards vaccination, both directly and indirectly through reduced susceptibility to vaccination-related myths [16].

Equally noteworthy was our finding that participants with low or medium SES had the highest health literacy, contrary to typical reported correlations [23]. In general population studies, lower SES and particularly lower educational attainment is consistently identified as the most important determinant of limited health literacy [34, 35]. Several factors may explain this discrepancy in the present study. First, the online survey design may have introduced selection bias, as digital participation requires basic digital literacy and internet access, which are more prevalent among individuals with higher SES [36, 37]. Consequently, participants with low or medium SES who completed the questionnaire may represent a positively selected subgroup with above-average health literacy skills. Second, the subjective, self-reported nature of the health literacy measure may have contributed to this finding, as self-assessed competence does not necessarily correspond to objectively measured skills [33]. Third, the quarantine-specific adaptation of the health literacy items may have captured situational competencies that differ from general health literacy, potentially narrowing the usual SES gradient. Despite this unexpected finding, respondents with higher SES were more willing to be vaccinated than those with low or medium SES, consistent with existing literature [8, 9].

Despite these heterogeneous results, future vaccination strategies must be tailored more specifically to target groups. For instance, younger populations should be addressed via low-threshold digital channels [38]. Meanwhile, culturally sensitive and multilingual information services are essential for people with a migration background to overcome potential linguistic and cultural barriers [31, 39, 40]. Promotion of health literacy should focus more on the ‘unsure middle’, which is particularly susceptible to misinformation. Finally, addressing social inequalities requires vaccination campaigns targeted at population groups with lower SES to reduce health disparities.

### Strengths and limitations

A key strength of the study lies in the large regional sample and the broad coverage across several survey waves, based on comprehensive data from health authorities on all IPs and CPs, which provides a unique population perspective. In addition, the consideration of health literacy as a key explanatory variable is particularly valuable, as this factor remains underrepresented in vaccination research. However, selection bias cannot be ruled out, as indicated by the distribution of health literacy and SES. People without an email address could not be contacted, and digital completion of the questionnaire required basic digital literacy and access to appropriate media. This may have led to the underrepresentation of certain groups, particular those with low digital literacy and/or limited access to digital media, such as the elderly. Consequently, the findings may not be fully generalizable to the broader population, and the true extent of vaccine hesitancy and its determinants may be underestimated. Health literacy data were also missing for around 600 participants, which may have introduced additional bias.

Beyond sampling issues, several measurement-related limitations must be considered. Because the data are cross-sectional, causal inferences cannot be established; the associations reported — for example between health literacy and vaccination willingness — should therefore be interpreted as correlational rather than directional. The information is self-reported and may be distorted by social desirability. For example, it was not possible to validate data on vaccinations. The use of language predominantly spoken at home as a proxy for migration background may not fully capture this construct. SES was assessed using education and professional qualifications only, without income data, which limits comparability with other studies using full SES indices. Furthermore, the dichotomization of SES into “high” versus “low/medium” may obscure potentially meaningful differences between low and medium SES groups and should be considered when interpreting the results. Health literacy was measured using items adapted from the validated HLS19-Q47 questionnaire and modified for the quarantine context, as used in the HLS-GER 2 study [23]. As the adapted version was not formally validated, comparability with studies using the original HLS19-Q47 or other standardized health literacy instruments may be limited. Finally, the regression model explains only 5.4% of the variance in vaccination willingness, indicating that important determinants were not captured. Variables such as political attitudes, trust in the media, or social norms have been identified as key factors influencing vaccination decisions in other studies and were not assessed in the present survey [41–43]. Their inclusion in future research could substantially improve the explanatory power of predictive models.

## Conclusion

In summary, this analysis shows high vaccination willingness within the study sample, with significant associations observed for age, migration background, a COVID-19-infection (IP), chronic diseases, SES, and health literacy. Taken into account that only 5.4% of the variance could be explained, the strongest association was found for quarantine as an infected person compared to a contact person, suggesting that direct experience with the disease may play a relevant role in vaccination decisions. Particularly vulnerable groups were younger adults, individuals with a migration background, and those with medium health literacy. The results underscore the need for target group-oriented, low-threshold, and culturally sensitive vaccination strategies, as well as measures to strengthen health literacy. Overall, this study helps explain vaccination behaviour in Germany and provides practical starting points for future pandemic management and prevention strategies. However, our findings should be verified in prospective longitudinal analyses to substantiate possible causal factors influencing vaccination willingness. On this basis, more targeted vaccination and education campaigns should be developed.

## Data Availability

The datasets used and/or analysed during the current study are available from the corresponding author on reasonable request.

## Abbreviations

OR: Odds ratio
CP: Contact person
IP: Infected person
SES: Socioeconomic status
HL: Health literacy
BMI: Body mass index
cm: Centimetres
kg: Kilograms
m: Metres
BMI: Body mass index
PCR: Polymerase chain reaction
aPR: Adjusted Prevalence Ratio
